# Deductive Verification of Floating-Point Java Programs in KeY

**DOI:** 10.1007/978-3-030-72013-1_13

**Published:** 2021-02-26

**Authors:** Rosa Abbasi, Jonas Schiffl, Eva Darulova, Mattias Ulbrich, Wolfgang Ahrendt

**Affiliations:** 8 Eindhoven University of Technology, Eindhoven, The Netherlands; 9 Aalborg University, Aalborg East, Denmark; 10 MPI-SWS, Kaiserslautern and Saarbrücken, Saarbrücken, Germany; 11 Karlsruhe Institute of Technology, Karlsruhe, Germany; 12 Chalmers University of Technology, Göteborg, Sweden

**Keywords:** Deductive Verification, Floating-point Arithmetic, Transcendental Functions.

## Abstract

Deductive verification has been successful in verifying interesting properties of real-world programs. One notable gap is the limited support for floating-point reasoning. This is unfortunate, as floating-point arithmetic is particularly unintuitive to reason about due to rounding as well as the presence of the special values infinity and ‘Not a Number’ (NaN). In this paper, we present the first floating-point support in a deductive verification tool for the Java programming language. Our support in the KeY verifier handles arithmetic via floating-point decision procedures inside SMT solvers and transcendental functions via axiomatization. We evaluate this integration on new benchmarks, and show that this approach is powerful enough to prove the absence of floating-point special values—often a prerequisite for further reasoning about numerical computations—as well as certain functional properties for realistic benchmarks.

### 1 Introduction

Deductive verification has been successful in providing functional verification for programs written in popular programming languages such as Java [4,23,41,49], Python [29], Rust [6], C [25,54], and Ada [19,50]. Deductive verifiers allow a user to annotate methods in a program with pre- and postconditions, from which they automatically generate verification conditions (VCs). These are then either proven directly by the verifier itself, or discharged with external tools such as automated (SMT) solvers or interactive proof assistants.

While deductive verifiers fully implement many sophisticated data representations (including heap data structures, objects, and ownership), support for floating-point numbers remains rather limited – solely Frama-C and SPARK offer automated support for floating-point arithmetic in C and Ada [32]. This state of affairs is at least partially a result of previous limitations in floating-point support in SMT solvers. Consequently, deductive verification has been used for floating-point programs only by experts with considerable manual effort [15,32]. This is unfortunate as it makes deductive verification unavailable for a large number of programs across many domains including embedded systems, machine learning, and scientific computing. With the increasing need for parallelization in code, scientific computing specifically has recently experienced algorithmic challenges for which formal methods may contribute to a solution [10,56].

One of the main challenges of floating-point arithmetic is its unintuitive behavior and the special values that the IEEE 754 standard [39] introduces. For instance, an overflow or a division by zero results in the special value (positive or negative) *infinity*, and not a runtime exception. Similarly, invalid operations like `sqrt(-1.0)` result in a *Not a Number* (NaN) value. These special values are problematic as seemingly straight-forward identities do not hold (`x == x` or `x * 0.0 == 0.0`). In addition, every operation on floating-point numbers potentially involves rounding, which compromises familiar rules like associativity and distributivity. Hence, reasoning support for writing correct floating-point programs is indispensable.

Abstract interpretation-based tools can prove the absence of runtime errors and special values [20,43], and bound roundoff errors due to floating-point's finite precision [11,21,26,36,57]. SMT decision procedures [18] or SAT-based model-checking [24,56], on the other hand, can prove intricate properties requiring bit-precise reasoning. However, these techniques and tools largely support only purely floating-point programs or program snippets, or analyze programs only up to a predefined depth of the call stack. General reasoning about real-world object-oriented programs, however, also requires support for features such as the (unbounded) heap, necessitating different analyses which need to be combined with floating-point reasoning.

Handling floating-points in a deductive verifier has unique advantages. First, the deductive verification approach already comes with the infrastructure for reasoning about complex control and data structures (like exception handling and heap). Second, it allows one to flexibly combine the verifier's symbolic execution reasoning with external decision procedures. Third, depending on the theory support, the verifier or external solver may also generate counterexamples of a property and thus help program debugging – something an abstract interpretation-based approach fundamentally cannot provide.

We report on adding floating-point support to the KeY deductive verifier, providing the first automated deductive floating-point support for the Java programming language. We focus mainly on proving the absence of the special values infinity and NaN. While these are helpful in certain circumstances, for most applications they signal an error. Hence, showing their absence is a prerequisite for further (functional) reasoning. That said, our extension also allows one to express and discharge arbitrary functional properties expressible in floating-point arithmetic, including bounds on roundoff errors for certain programs, and bounds on differences between two similar floating-point programs

We exploit both KeY's symbolic execution and external SMT support. On the one hand, we handle arithmetic operations by relying on a combination of

KeY's symbolic execution to handle the heap and SMT based decision procedures to handle the floating-point part of the VCs. On the other hand, we support transcendental functions via axiomatization in the KeY prover itself.

Transcendental functions such as sine are a common feature in numerical programs, but are not supported by floating-point decision procedures. We explore two ways of supporting them soundly but approximately, by encoding them as axiomatized uninterpreted function symbols once directly in the SMT queries, and once in additional calculus rules in KeY. Our evaluation shows that even though such reasoning is approximate, it is nonetheless sufficient to prove the absence of special values in many interesting programs.

We evaluate KeY's floating-point support on a number of real-world floating-point Java programs. Our benchmark set allows us to evaluate recent progress in SMT floating-point support in Z3 [28], CVC4 [8] and MathSAT [22] on yet unseen benchmarks. For instance, we observe that quantifiers are challenging even if they do not affect satisfiability of SMT queries. Our benchmarks are openly available, and we expect our insights to be useful for further solver development.

*Contributions* In summary, we make the following contributions:

– we implement and evaluate the first automated deductive verification of floating-point Java programs by combining the strength of rule based and SMT based deduction;
– we collect a new set of challenging real-world floating-point benchmarks in Java (available at https://gitlab.mpi-sws.org/AVA/key-float-benchmarks/);
– we compare different SMT solvers for discharging floating-point VCs on this new set of benchmarks;
– and we develop novel automated support for reasoning about transcendental functions in a deductive verifier.

### 2 Background

#### 2.1 Introduction to KeY

KeY [4] is a platform for deductive verification of Java programs, working at a source code level. The input is a Java program annotated in the Java Modeling Language (JML) [45], encouraging a *Design by Contract* ([46,51]) approach to software development. The user specifies the expected behavior of Java classes with *class invariants* that the program has to maintain at critical points. Methods are specified with *method contracts*, consisting mainly of pre- and postconditions, with the understanding that if the precondition holds when the method is called, the postcondition has to hold after the method returns.

After loading an annotated program, KeY translates it to a formula in Java Dynamic Logic [4] (JavaDL), an instance of Dynamic Logic [37] which enables logical reasoning about Java programs. Logical rules are provided for the translation of programs into first-order logic, and for closing the resulting *goals*, or proof obligations. KeY is semi-interactive in that it allows manual rule application, while also offering powerful built-in automation and macros. In addition, it is also possible to translate an open goal into SMT-LIB format [9] and call an external SMT solver. For specific theories, SMT solvers can be much more efficient than KeY's own automation. This makes it possible to prove some goals, which depend on SMT supported theories, by using an SMT solver, while others are proved internally, using KeY's own automation.

#### 2.2 Floating-Point Arithmetic in Java

In the following, we summarize some central characteristics of Java floating-point numbers, loosely following [53]. Each *normal* floating-point number $x$ can be represented as a triplet $(s, m, e)$, such that $x = (-1)^s * m * 2^e$, where $s \in \{0, 1\}$ is the *sign*, $m$ (called *significand*) is a binary fixed-point number with one digit before the radix point and $p-1$ digits after the radix point (note that $0 \leq m < 2$), and $e$ (*exponent*) is an integer such that $e_{min} \leq e \leq e_{max}$. Java supports two floating-point formats (both in base 2): `float` ('single') precision with $p = 24$, and minimal and maximal exponent $e_{min} = -126, e_{max} = 127$ and `double` precision with $p = 53, e_{min} = -1022, e_{max} = 1023$.

Whenever the result of a computation cannot be exactly represented with the given precision, it is rounded. IEEE 754 defines various rounding modes, of which Java only supports *round to nearest, ties to even.* Rounding is exact, as if one would first compute the ideal real number, and round afterwards.

The triple representation gives us two zeros, $+0$ and $-0$, represented by $(0, 0, 0)$ and $(1, 0, 0)$, respectively. If the absolute value of the ideal result of a computation is too small to be representable as a floating-point number of the given format, the resulting floating point number is $+0$ or $-0$. In addition, there are three special values, $+\infty$, $-\infty$, and NaN (Not a Number). If the absolute value of the ideal result of a computation is too big to be representable as a floating-point number of the given format, the result is $+\infty$ or $-\infty$. Also, division by zero will give an infinite result (e.g., $7.13/+0 = +\infty$). Computing further with infinity may give an infinite result (e.g., $+\infty + +\infty = +\infty$), but may also result in the additional 'error value' NaN (e.g., $+\infty - +\infty = \text{NaN}$). Due to the presence of infinities and NaN, floating-point operations do *not* throw Java exceptions.

By default, the Java virtual machine is allowed to make use of higher-precision formats provided by the hardware. This can make computation more accurate, but it also leads to platform dependent behaviour. This can be avoided by using the `strictfp` modifier, ensuring that only the single and double precision types are used. This modifier ensures portability.

### 3 Floating-Point Support in KeY

#### 3.1 Arithmetics

In order to be able to specify and verify programs containing floating-point numbers, we made several extensions to the KeY tool. First, we added the `float`

Listing 1.1: The Rectangle.scale benchmark

```
/*@ public normal_behavior
  @  requires \fp_nice(arg0.x) && \fp_nice(arg0.y)
  @    && \fp_nice(arg1) && \fp_nice(arg2);
  @  ensures !\fp_nan(\result.x) && !\fp_nan(\result.y) &&
  @   !\fp_nan(\result.width) && !\fp_nan(\result.height);
  @ also
  @ public normal_behavior
  @  requires -5.53 <= arg0.x && arg0.x <= -3.38 &&
  @    -5.53 <= arg0.y && arg0.y <= -3.38 &&
  @    3.1 < arg0.width && arg0.width <= 3.7332 &&
  @    3.0000001 < arg0.height && arg0.height <=4.0004 &&
  @    3.0003001 < arg1 && arg1 <= 4.0024 &&
  @    -6.4000003 < arg2 && arg2 <= 3.0001;
  @  ensures !\fp_nan(\result.x) && !\fp_nan(\result.y)&&
  @   !\fp_nan(\result.width) &&!\fp_nan(\result.height);
  @*/
public Rectangle scale(Rectangle arg0, double arg1, double arg2){
  Area v1 = new Area(arg0);
  AffineTransform v2 = AffineTransform.getScaleInstance(arg1, arg2);
  Area v3 = v1.createTransformedArea(v2);
  Rectangle v4 = v3.getRectangle2D();
  return v4;
}
```

and `double` types to the KeY type system, together with an enum type for the different rounding modes of the IEEE 754 Standard.

We further introduced functions and predicate symbols to formalize operations (+, ∗, . . . ) and comparisons (<, ==, . . . ) on floating-point expressions. The translation supports both code with and without the `strictfp` modifier. However, since the actual precision of non-strictfp operations is not known, the function symbols remain uninterpreted. We extended KeY's parser to correctly handle programs and annotations containing floating-point numbers, and added logic rules for translating floating-point expressions from Java or JML to JavaDL.

As an example, Listing 1.1 shows JML specifications of our `Rectangle` benchmark that contains floating-point literals and makes use of the `fp_nan` and `fp_nice` predicates. `fp_nan` states that a floating-point expression is NaN and `fp_nice`, which is shorthand for "not infinity and not NaN", states that a floating-point expression is not NaN or infinity. The `scale` method contains two contracts that are checked separately, ensuring that the class fields of a scaled rectangle object are not NaN, considering different preconditions. For the first contract, the SMT solver produces a counterexample. In the second, we bound inputs by concrete ranges that we picked arbitrarily and get the valid result. In practice, such ranges would come from the context, e.g. from the kind of rectangles that appear in an application, or from known ranges of sensor values.

Concerning discharging the resulting proof obligations, there were two main ways to consider. One is to create a floating-point theory within KeY by adding axioms and deduction rules, so that the desired properties can be proven in KeY's sequent calculus. The other way is to translate the proof obligations from JavaDL to SMT-LIB and call an external SMT solver. While the KeY approach traditionally favors conducting proofs within KeY, for this work, we partially deviated from this way in order to harness the greater experience and efficiency of SMT solvers when it comes to floating-point arithmetic. Our approach attempts to get the best of both worlds by distinguishing between basic floating-point arithmetic, i. e., elementary operations and comparisons, and more complex functions which do not have an SMT-LIB equivalent (e. g., the transcendental functions), or where the SMT-LIB function is not usefully implemented by current SMT solvers (see Section 3.2.B).

Elementary operations and comparisons get translated to the corresponding SMT-LIB functions. In SMT-LIB, all floating-point computations conform to the IEEE 754 Standard. Therefore, only Java programs with the `strictfp` modifier can be directly translated to SMT-LIB without loss of correctness.

We developed a translation from KeY's floating-point theory to SMT-LIB. In order to integrate it into KeY, we also overhauled the existing translation from JavaDL to SMT-LIB to create a new, more modular framework, which now supports all the features of the original translation, e. g., heaps and integer arithmetic, but also floating-point expressions at the same time.

Floating-point intricacies sometimes require extra caution. For example, there are two different notions of equality for floats: bitwise equality and IEEE754 equality. Our implementation ensures these are distinguished correctly, and that the specification language remains intuitive for a developer to use.

Using the translation to SMT-LIB, we can specify and prove two classes of properties in KeY: The absence of special values is specified using the `fp_nan` and `fp_infinite` predicates (or the `fp_nice` equivalent). Furthermore, one can specify *functional* properties that are expressible in floating-point arithmetic, e.g. one can compare the result of a computation against the result of a different program which is known to produce a good result or a reference value.

#### 3.2 Transcendental Functions

Floating-point decision procedures in SMT solvers successfully handle programs consisting of arithmetic and square root operations. Many numerical real-world programs, however, include transcendental functions such as `sin` and `cos`. In Java programs, these functions are implemented as static library functions in the class `java.lang.Math`.

Unlike arithmetic operations, transcendental functions are much more loosely specified by the IEEE 754 Standard—only an upper bound on the roundoff error is given. Libraries are thus free to provide different implementations, and even tighter error bounds. Exact reasoning in the same spirit as floating-point arithmetic would thus have to encode a specific implementation. Given that these implementations are highly optimized, this approach would be arguably complex.

We observe, however, that such exact reasoning about transcendental functions is often not necessary and a sound approximate approach is sufficient and efficient.

In this section, we introduce an axiomatic approach for reasoning about programs containing transcendental functions. We observe that with the flexibility of deductive verification and KeY itself, we can instantiate it in two different ways. We encode transcendental functions as uninterpreted functions and axiomatize them in the SMT queries. Alternatively, we encode these axioms in KeY as logical inference rules.

**(A) Axiomatization in SMT** We encode library functions as uninterpreted functions and include a set of axioms in the SMT-LIB translation for each method that is called in a benchmark. That is, we extended KeY such that when a transcendental function exists in the proof obligation, its definition alongside all the axioms for that function are added to the translation.

For the axiomatization of transcendentals, we did *not* add rules that expand to a definition or allow a repeated approximation of the function value (like expansion into a Taylor series). Instead, we added a number of lemmata encoding interesting properties related to special values. For instance, the following axiom states that if the input to the `sin` function is not a NaN or infinity, then the returned value of `sin` is between $-1.0$ and $1.0$:

```
(assert (forall ((a Float64)) (=>
  (and (not (fp.isNaN a)) (not (fp.isInfinite a)))
  (and (fp.leq (sinDouble a) (fp #b0 #b01111111111 #b0000...000000))
       (fp.geq (sinDouble a) (fp #b1 #b01111111111 #b0000...000000))))))
```

Note that this implies that the result is not a NaN or infinity. The other axioms are similar in spirit, so we do not list them.

These axioms are expressed as quantified floating-point formulas and capture high-level properties of library functions complying with the specifications in the IEEE 754 Standard. Clearly, since we do not have the actual implementations of these functions, we are not able to prove arbitrary properties. However, such an axiomatization is often sufficient to check for the (absence of) special values, i.e. NaN and infinity, as our experiments in Section 4.4 show.

**(B) Taclets in KeY** Reasoning about quantified formulas in SMT is a long-lasting challenge [34]. We have also observed in our experiments with only arithmetic operations (Section 4.3) that SMT solvers struggle with quantifiers in combination with floating-points. We have therefore implemented an alternative approach encoding the axioms not in the SMT queries, but instead as deductive inference rules (so-called taclets) in KeY.

The rules encode the same logical information as the universally quantified assertions that we add in SMT-LIB (and where we leave the choice of instantiations entirely to the SMT/SAT solver). With our taclet approach, we instantiate a quantifier (only) to one's needs. We note that for proving a property correct, this results in a correct (under)approximation. However, the prize for achieving

| | Benchmark Details | | | | Automode Statistics | | | |
|---|---|---|---|---|---|---|---|---|
| benchmark | # classes | # method calls | # arith. ops | library functions | # goals closed by KeY | # goals to be closed externally | # rules applied | automode time (s) |
| Complex.add (2) | 1 | 0 | 2 | - | 3 / 3 | 1 / 4 | 185 / 286 | 0.7 / 0.2 |
| Complex.divide (2) | 1 | 0 | 11 | - | 10 / 8 | 2 / 8 | 483 / 625 | 0.7 / 0.8 |
| Complex.compare | 1 | 0 | 2 | - | 3 | 2 | 216 | 0.2 |
| Complex.reciprocal (2) | 1 | 1 | 6 | - | 1 / 1 | 2 / 2 | 402 / 406 | 0.4 / 0.5 |
| Circuit.impedance | 2 | 1 | 3 | - | 1 | 4 | 360 | 0.5 |
| Circuit.current (2) | 2 | 3 | 14 | - | 11 / 11 | 4 / 1 | 1267 / 1238 | 4.0 / 4.1 |
| Matrix2.transposedEq | 1 | 3 | 3 | - | 3 | 1 | 735 | 0.9 |
| Matrix3.transposedEq | 1 | 4 | 34 | - | 3 | 1 | 1786 | 5.1 |
| Matrix3.transposedEqV2 | 1 | 4 | 34 | - | 3 | 1 | 1796 | 5.4 |
| Rectangle.scale (2) | 3 + 1 | 23 | 22 | - | 32 / 32 | 32 / 16 | 5990 / 5617 | 18.4 / 14.5 |
| Rotate.computeError | 1 + 1 | 6 | 26 | - | 108 | 8 | 3693 | 74.2 |
| Rotate.computeRelErr | 1 + 1 | 6 | 28 | - | 120 | 8 | 3898 | 79.6 |
| FPLoop.fploop | 1 | 0 | 1 | - | 2 | 4 | 99 | 0.1 |
| FPLoop.fploop2 | 1 | 0 | 1 | - | 2 | 4 | 99 | 0.1 |
| FPLoop.fploop3 | 1 | 0 | 1 | - | 2 | 4 | 99 | 0.1 |
| Cartesian.toPolar | 2 + 1 | 3 | 6 | sqrt, atan | 1 | 4 | 438 | 0.5 |
| Cartesian.distanceTo | 1 + 1 | 1 | 5 | sqrt | 2 | 1 | 191 | 0.1 |
| Polar.toCartesian | 2 + 1 | 3 | 4 | cos, sin | 1 | 2 | 364 | 0.5 |
| Circuit.instantCurrent | 2 + 1 | 14 | 23 | sqrt, atan, cos | 17 | 2 | 1686 | 14.1 |
| Circuit.instantVoltage | 1 + 1 | 1 | 4 | cos | 0 | 2 | 138 | 0.1 |

Table 1: Benchmark details and KeY automode statistics, time is measured in seconds more closed proofs and shorter running times is that for disproving a property, not considering all possible quantifier instantiations may lead to spurious counterexamples, i.e., false positives.

A heuristic strategy applies the rules automatically using the occurrences of transcendentals as instantiation triggers. However, instantiating the axioms too eagerly, considerably increases the number of open goals, which is why we assume that the user selects the axioms to apply manually (and did so in the experiments). After the application the proof obligation can either be closed, i.e proven, by KeY automatically, or be given to the SMT solver as before for final solving.

Currently, the set of axioms (in the SMT-LIB translation and as taclets in KeY) only contains axioms for the transcendental functions occurring in our benchmarks. So far we have 10 axioms; however, adding more axioms (also for further transcendentals like exponentiation or logarithm) is straightforward. The full set of axioms is included in the Appendix of the technical report [3].

## 4 Evaluation

### 4.1 Benchmark Programs

We collected a set of existing floating-point Java programs representing real-world applications in order to evaluate the feasibility and performance of KeY's floating-point support.

The left half of Table 1 provides an overview of our benchmarks. Each benchmark consists of one method, which is composed of arithmetic operations

Listing 1.2: The Circuit.instantCurrent benchmark

```
public class Circuit {
double maxVoltage, frequency, resistance, inductance;
// ...

/*@ public normal_behavior
  @ requires 1.0 < this.maxVoltage && this.maxVoltage < 12.0 &&
  @  1.0 < this.frequency && this.frequency < 100.0 &&
  @  1.0 < this.resistance && this.resistance < 50.0 &&
  @  0.001 < this.inductance && this.inductance < 0.004 &&
  @  0.0 < time && time < 300.0;
  @ ensures !\fp_nan(\result) && !\fp_infinite(\result);
  @*/
public double instantCurrent(double time) {
  Complex current = computeCurrent();
  double maxCurrent = Math.sqrt(current.getRealPart() * current.getRealPart() +
    current.getImaginaryPart() * current.getImaginaryPart());
  double theta = Math.atan(current.getImaginaryPart() / current.getRealPart());
  return maxCurrent * Math.cos((2.0 * Math.PI * frequency * time) + theta);
}}
```

and method calls to potentially other classes. The invocations of methods from `java.lang.Math` (e.g. `Math.abs`) are marked by "+1" in Table 1; these are resolved by inlining the method implementation. For benchmarks that contain calls to transcendental functions and square root, the called functions are listed; these are handled by our axiomatization. We include `sqrt` in this list, as we have observed that exact support can be expensive, so it may be advantageous to handle `sqrt` axiomatically. Benchmarks `Rectangle`, `Circuit`, `Matrix3` and `Rotation` are partially shown in Listings 1.1, 1.2, 1.3 and 1.4 respectively.

Each benchmark also includes a JML contract that is to be checked. For some methods, we specify two contracts (marked by "(2)" in the first column of Table 1), each serving as an independent benchmark. The contracts for most of these benchmarks check that the methods do not return a special value i.e infinity and/or NaN, the preconditions being that the variables are not themselves special values and possibly are bounded in a given range. For the `Matrix`, `FPLoop` and `Rotate` benchmarks, we check a *functional* property (see Section 4.3). `FPLoop`, which has three contracts, additionally shows how to specify floating-point loop behavior using loop invariants.

#### 4.2 Proof Obligation Generation

To reason about the contract of a selected benchmark, we apply KeY, which generates proof obligations or 'goals'. Some of these goals (heap-related) are closed by KeY automatically. The remaining open goals are closed by either SMT solvers with floating-point support directly (Section 3.1 and Section 3.2.A), or with a combination of transcendental KeY taclets and floating-point SMT solving (Section 3.2.B).

Columns 6 and 7 in Table 1 show the number of proof obligations closed by KeY directly and to be discharged by external solvers, respectively. The next two columns show the number of taclet rules that KeY applied in order to close its goals, and the time this takes. For benchmarks with two contracts we show the respective values separated by '/'.

We run our experiments on a server with 1.5 TB memory and 4x12 CPU cores at 3 GHz. However, KeY runs single-threadedly and does not use more than 8GB of memory.

For our set of benchmarks, the symbolic execution process is fully automated. Note that the machinery can deal with loop invariants, if they are provided. Loop invariant generation is, however, particularly challenging for floating-points due to roundoff errors [27, 40], and a research topic in itself.

#### 4.3 Evaluation of SMT Floating-Point Support

Previous work [32] reported that SMT support for floating-point arithmetic is rather limited. However, with recent advances [18], we evaluate the situation again. Most benchmarks used to evaluate SMT solvers' decision procedures [1] aim to check (individual) specialized (corner case) properties of floating-point arithmetic. The proof obligations generated from our set of benchmarks are complementary in that they are more arithmetic heavy, while nonetheless relying on accurate reasoning about special values and functional properties.

For each open goal not automatically closed, KeY generates one SMT-LIB file that is fed to the solvers for validation. We compare the performance of the three major SMT solvers with floating-point support CVC4 [8] (version 1.8, with the SymFPU library [18] enabled), Z3 (4.8.9) [28] and MathSAT (5.6.3) [22]. For this we set a timeout of 300s for each proof obligation. While KeY is able to discharge proof obligations in parallel, for our experiments, we do so sequentially to maintain comparability.

KeY's default translation to SMT includes quantifiers. These quantifications are not related to floating-point arithmetic, but are used to logically encode important properties of the Java memory model, like the type hierarchy and the absence of dangling references on any valid Java heap. If we reason about floating-point problems in isolation, they are not needed, but if we want to consider Java verification more holistically with questions combining aspects of heap and floating point reasoning, they become essential. We manually inspected that the proof obligations without our axiomatized treatment of transcendental functions do not depend on these properties and investigate the quantifier support by including or removing them from the SMT translations. We do not report results with quantifiers for MathSAT, since it does not support them.

Table 2 summarizes the results of our experiments. Column 4 shows the number of expected valid or invalid goals for all benchmarks. For each solver we show the number of goals that each solver can validate or invalidate, together with the average time (in seconds) needed. The goals resulting in timeout were

| index | experiment | quantified axioms | # goals | CVC4 | | Z3 | | MathSAT | |
|---|---|---|---|---|---|---|---|---|---|
| | | | | # goals decided | avg. | # goals decided | avg. | # goals decided | avg. |
| 1 | valid | ✓ | 80 | 79 | 4.1 | 25 | 18.4 | - | - |
| 2 | contracts | ✗ | 80 | 79 | 4.0 | 52 | 35.0 | 80 | 8.8 |
| 3 | invalid | ✓ | 9 | 0 | 3.4 | 0 | 3.4 | - | - |
| 4 | contracts | ✗ | 9 | 8 | 36.7 | 7 | 27.6 | 9 | 3.9 |
| 5 | axioms in SMT | ✓ | 10 | 9 | 33.2 | 4 | 63.4 | - | - |
| 6 | axioms as taclets | ✗ | 10 | 10 | 33.4 | 5 | 74.2 | 8 | 0.9 |
| 7 | fp.sqrt | ✗ | 7 | 7 | 46.2 | 1 | 23.5 | 5 | 0.4 |
| 8 | axiomatized sqrt | ✗ | 7 | 5 | 2.4 | 5 | 282.8 | 5 | 5.7 |

Table 2: Summary of valid / invalid goals correctly decided and average running times of each solver for the SMT translations with and without quantified axioms

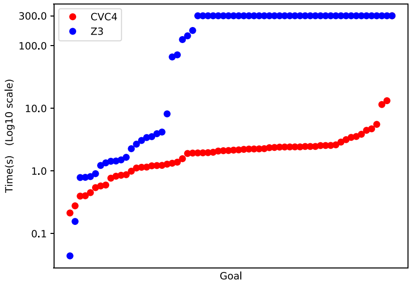

Fig. 1: Runtimes for valid goals with SMT translations *with* quantifiers

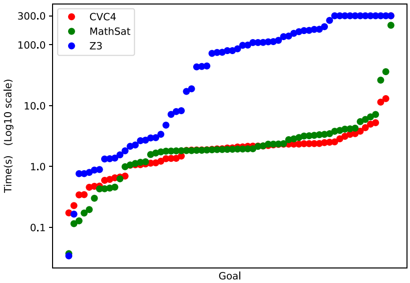

Fig. 2: Runtimes for valid goals with SMT translations *without* quantifiers excluded from the computation of the average time. Column 3 shows whether the SMT queries include quantifiers or not.

Rows 1 and 2 of Table 2 show the results for benchmarks with valid contracts. This experiment thus represents the common behavior of KeY, whose main goal is to *prove* contracts correct. Rows 3 and 4 of Table 2 demonstrate the results for benchmarks with invalid contracts, i.e. for those we expect a counterexample for at least one of the goals. The Appendix of the technical report [3] contains the detailed results for each experiment separated by benchmark. Figure 1 and Figure 2 show a more detailed view of the solvers' running time for the valid benchmarks. The x-axis shows the number of open goals that are discharged by the SMT solvers, sorted by running time for each solver individually. The $k$-th point of one graph shows the minimum running time needed by the solver to close each of the $k$ fastest goals. Note that each solver may have different goals which are its $k$ fastest. The y-axis shows the time on a logarithmic scale.

We conclude that in the presence of quantified axioms and floating-point arithmetic solvers' performance deteriorate for both valid and invalid goals. In particular, none of the solvers is able to find counterexamples for any of the invalid goals. However, when the quantified axioms are removed from the

SMT translations, their performance improves. For valid contracts, CVC4 and MathSAT perform better than Z3, in terms of both number of goals validated and the running time per goal. In particular, MathSAT is able to prove all goals. However, the running time performance of CVC4 is better than MathSAT's. For invalid contracts, solvers are able to produce the expected counterexamples at least partially. Particularly, MathSAT has a better performance than CVC4 and Z3 in terms of both running time and the number of proof obligations for which it can produce counterexamples.

We conducted another experiment on our `Rectangle.scale` benchmark to assess the solvers' sensitivity to various changes, applied to the benchmark's contract or its implementation. We considered modifications such as reducing the number of classes while keeping the same functionality, having tighter and larger bounds for variables, reducing the number of arithmetic operations etc. The details of this experiment can be found in the Appendix of the technical report [3]. In summary, solvers' performance seems to be sensitive to slight innocuous looking changes such as the number of classes involved and variable bounds. For example, constraining `arg2` in the original benchmark more tightly allows CVC4 to validate all goals (1 more). This behavior could be potentially exploited by e.g. relaxing a variable's bounds.

*Proving Functional Properties* Listings 1.3 and 1.4 show examples of functional properties that are expressible in floating-point arithmetic and that KeY can handle. The verification results are included in rows 1 and 2 of Table 2, for more details see the Appendix of the technical report [3].

For `Matrix`, we check that the determinants of a matrix and its transpose are equal. Note that this property holds trivially under real arithmetic, but not necessarily under floating-points. After feeding `transposedEq` (which uses the `determinant` method) and its contract to KeY, increasing the default timeout sufficiently and discharging the created goal, CVC4 generates a counterexample in 170.2s seconds and MathSAT in 16.2s. Z3 times out after 30 minutes. By feeding `transposedEqV2` (which uses the `determinantNew` method) to KeY, CVC4 validates the contract in 1.1s, MathSAT in 3.9s and Z3 times out again. One thing worth noting is that the way programs are written can greatly influence the computational complexity needed to reject or verify the contract. This is evident from the fact that slightly modifying the order of operations (using `determinantNew` instead) substantially reduces verification time and changes the verification result for MathSAT and CVC4.

For `Rotate`, we check that the difference between an original vector and the one that is rotated four times by 90 degrees, must not be larger than 1.0E-15. We also verified the same bound for the relative difference (by exploiting another method and contract) for this benchmark. The constant `cos90` in Listing 1.4 is not precisely 0.0 to account for rounding effects in the computation of the cosine. `FPLoop` includes three loops, for which the contracts check that the return value is bigger than a given constant.

Though not always very fast, these examples show that verification of functional floating-point properties is viable.

Listing 1.3: The Matrix3 benchmark

```
public class Matrix3 {
  double a, b, c, d, e, f, g, h, i; //The matrix: [[a b c],[d e f],[g h i]]
  double det;
  // method transpose not shown double determinant() {
    return (a * e * i + b * f * g + c * d * h) -
      (c * e * g + b * d * i + a * f * h);
  }
  double determinantNew() {
    return (a * (e * i) + (g * (b * f) + c * (d * h))) -
      (e * (c * g) + (i * (b * d) + a * (f * h)));
  }
  /*@ ensures \fp_normal(\result) ==> (\result == det); @*/
  double transposedEq() {
    det = determinant();
    return transpose().determinant();
  }
  /*@ ensures \fp_normal(\result) ==> (\result == det); @*/
  double transposedEqV2() {
    det = determinantNew();
    return transpose().determinantNew();
  }
}
```

Listing 1.4: The Rotation benchmark

```
public class Rotation {
  final static double cos90 = 6.123233995736766E-17;
  final static double sin90 = 1.0;

// rotates a 2D vector by 90 degrees
  public static double[] rotate(double[] vec) {
    double x = vec[0] * cos90 - vec[1] * sin90;
    double y = vec[0] * sin90 + vec[1] * cos90;
    return new double[]{x, y};
  }
  /*@  requires (\forall int i; 0 <= i && i < vec.length;
    @   \fp_nice(vec[i]) && vec[i] > 1.0 && vec[i] < 2.0) && vec.length == 2;
    @  ensures  \result[0] < 1.0E-15 && \result[1] < 1.0E-15;
    */
  public static double[] computeError(double[] vec) {
    double[] temp = rotate(rotate(rotate(rotate(vec))));
    return new double[]{Math.abs(temp[0] - vec[0]), Math.abs(temp[1] - vec[1])};
  }
}
```

#### 4.4 Evaluation of Support for Transcendental Functions in KeY

We evaluated the two approaches from Section 3.2.A on our set of benchmarks; rows 5 and 6 in Table 2 summarize the results. (The detailed results of these experiments are included in the Appendix of the technical report [3].) Note that both approaches are fully automated.

We conclude that the SMT solvers perform better when the axiomatization is applied at the KeY level. When axioms for transcendental functions are added to the SMT-LIB translation directly Z3 validates 4 out of 10 goals. With the axiomatization at the KeY level, solvers are able to validate more goals (with quantified formulas removed from the SMT translations), e.g. Z3 is able to validate 5 goals and CVC4 can validate all. Therefore, it is preferable to apply them on the KeY side via taclet rules.

All the solvers we have used in this work comply with the IEEE 754 standard and therefore have bit-precise support for the square root function. They provide bit-precise reasoning by effectively encoding the behavior of floating-point circuits over bitvectors (which is naturally expensive), together with different heuristics and abstractions to speed up solving time. However, depending on the property, we do not always need bit-precise reasoning, so we propose handling the square root function with the same taclet-based axiomatization as introduced in Section 3.2.B.

To this end, we conducted an experiment on the benchmarks containing `sqrt`, comparing the approach from Section 3.2.B (adding the necessary axioms, resp. taclet rules) to using the square root implemented in SMT solvers (`fp.sqrt`). We chose to include only axioms specified in or inferred from the IEEE 754 standard (e.g. if the argument of the square root function is NaN or less than zero, then the square root results in NaN). The full set of axioms that we used is included in the Appendix of the technical report [3].

Rows 7 and 8 in Table 2 summarize the results for this experiment; the detailed results are included in the Appendix of the technical report [3]. We observed that for two out of the three benchmarks, the average running time of all solvers decreases using the axiomatized square root. Furthermore, Z3 is able to reason about more proof obligations with the axiomatized version. However, the success of this approach depends on the axioms added to KeY and may not always work if we do not have suitable axioms. For example, for the `Circuit.instantCurrent` benchmark (Listing 1.2), using the axiomatized square root, CVC4 is not able to validate the contract, but with `fp.sqrt` the contract is validated.

In summary, treating `sqrt` axiomatically can result in shorter solving times than performing bit-precise reasoning, but the approach may not always succeed when the axioms are not sufficient to prove a particular property.

#### 4.5 Discussion and insights

The experiments show that highly automated floating point program verification is viable for relevant properties (handling of special values and some functional properties), up to a certain level of complexity (given by the SMT solvers). The choices of which parts of a proof obligation are delegated to SMT, and how they are translated to SMT, are crucial for achieving effective and efficient program verification. Arithmetic operations proved to be more efficiently dealt with by delegation to SMT, whereas for transcendental functions, axiomatization and rule based treatment in the theorem prover, outside the SMT solver, performs clearly better.

### 5 Related Work

Our implementation uses the floating-point SMT-LIB theory [17], which however does not handle transcendental functions, as their semantics is (library) implementation dependent. Some real-valued automated solvers do handle transcendental functions [5, 33], but to the best of our knowledge, the combination of floating-points and reals in SMT solvers is still severely limited.

None of the existing deductive verifiers support floating-point transcendental functions automatically. The Why3 deductive verification framework [30] has support for floating-point arithmetic, with front-ends for the C and Ada programming languages through Frama-C [25] and SPARK [19, 32], respectively. Why3 has back-end support for different SMT solvers, as well as interactive proof assistants like Coq. Until recently, Why3 would discharge still many interesting floating-point problems with help of Coq, relying on significant user interaction. In later work [32] (in the context with floating-point verification for Ada programs), Why3 can achieve a higher degree of automation. Note, however, that the user is still required to add code assertions as well as 'ghost code' to a significant extent.

The Boogie intermediate verification language [47] also supports floating-point expressions, and targets Z3 for discharging proof obligations. In the Boogie community, it was observed that writing a specification in Boogie leads to decreases in SMT solver performance when compared to writing the goal in SMT-LIB directly, probably due to an inherent mixing of theories when using Boogie [2]. This matches our own experiences, and separation of theories should be considered an important task for the further development of floating-point verification.

Other deductive verifiers for Java have only rudimentary support for floating-points. Verifast [41] treats floating-point operations as if they were real values, and OpenJML [23] parses programs with floating-point operations, but essentially treats `float` and `double` as uninterpreted sorts.

The Java category of verification competition SV-COMP [12] contains a number of benchmarks that make use of floating-point variables. However, the focus of these benchmarks is usually not on arithmetical properties of expressions, but on the completeness of the Java language support. Amongst the participants of SV-COMP 2020, the Symbolic (Java) Pathfinder (SPF) [55] (and various extensions) and the Java Bounded Model Checker (JBMC) [24] support floating-point arithmetic. Besides being limited to exploring the state space up to a bounded depth, their constraint languages do not support quantifiers and abstracting of method calls—which are features that we have used in this work.

Floating-point arithmetic has also been formalized in several interactive theorem provers [16, 31, 42]. While one can prove intricate properties about floating-point programs [14,15,38], proofs using interactive provers are to a large part manual and require significant expertise.

Abstract interpretation based techniques can show the absence of special values in floating-point code fully automatically, and several abstract domains which are sound with respect to floating-point arithmetic exist [20,43]. While the analysis itself is fully automated, applying it successfully to real-world programs in general requires adaptation to each program analyzed by end-users, e.g. the selection of suitable abstract domains or widening thresholds [13].

Besides showing the absence of special values, recent research has developed static analyses to bound floating-point roundoff errors [26, 35, 48, 52, 57]. These analyses currently work only for small arithmetic kernels and the tools in particular do not accept programs with objects.

Dynamic analyses generally scale well on real-world programs, but can only identify bugs (when given failure-triggering input), rather than proving correctness for *all* possible inputs. Executing a floating-point program together with a higher-precision one allows one to find inputs which cause large roundoff errors [11,21,44]. Ariadne [7] uses a combination of symbolic execution, real-valued SMT solving and testing to find inputs that trigger floating-point exceptions, including overflow and invalid operations. Our work subsumes this approach as the SMT solvers that we use can directly generate counterexamples, but more importantly, KeY is able to prove the absence of such exceptions.

### 6 Conclusion

By joining the forces of rule-based deduction and SAT-based SMT solving, we presented the first working floating-point support in a deductive verification tool for Java and by that close a remaining gap in KeY to now support full sequential Java. Our evaluation shows that for specifications dealing with value ranges and absence of NaN and infinity, our approach can verify realistic programs within a reasonable time frame. We observe that the MathSAT and CVC4 solver's floating-point support scales sufficiently for our benchmarks, as long as the queries do not include any quantifiers, and that our axiomatized approach for handling transcendental functions is best realized using calculus rules in KeY's internal reasoning engine. While our work is implemented within the KeY verifier, we expect our approach to be portable to other verifiers.

**Acknowledgements**

This research was partially funded by the Deutsche Forschungsgemeinschaft (DFG, German Research Foundation) project 387674182. The authors would like to thank Daniel Eddeland, who together with co-author W. Ahrendt performed prestudies which impacted the current work.

## References

[1] QF$$\_$$FP SMT benchmarks. https://clc-gitlab.cs.uiowa.edu:2443/SMT-LIB-benchmarks/QF_FP (2019)

[2] Slow verification of programs combining multiple floating point values (Github issue) (2019 (accessed May 11, 2020)), https://github.com/boogie-org/boogie/issues/109

[3] Abbasi, R., Schiffl, J., Darulova, E., Ulbrich, M., Ahrendt, W.: Deductive Verification of Floating-Point Java Programs in KeY. CoRR **abs/2101.08733** (2021)

[4] Ahrendt, W., Beckert, B., Bubel, R., Hähnle, R., Schmitt, P.H., Ulbrich, M. (eds.): Deductive Software Verification - The KeY Book - From Theory to Practice, LNCS, vol. 10001. Springer (2016)

[5] Akbarpour, B., Paulson, L.C.: MetiTarski: An Automatic Theorem Prover for Real-Valued Special Functions. Journal of Automated Reasoning **44**(3) (2010)

[6] Astrauskas, V., Müller, P., Poli, F., Summers, A.J.: Leveraging Rust Types for Modular Specification and Verification. In: Object-Oriented Programming Systems, Languages, and Applications (OOPSLA) (2019)

[7] Barr, E.T., Vo, T., Le, V., Su, Z.: Automatic Detection of Floating-point Exceptions. In: Principles of Programming Languages (POPL) (2013)

[8] Barrett, C., Conway, C.L., Deters, M., Hadarean, L., Jovanovi’c, D., King, T., Reynolds, A., Tinelli, C.: CVC4. In: Computer Aided Verification (CAV) (2011), snowbird, Utah

[9] Barrett, C., Stump, A., Tinelli, C., et al.: The SMT-LIB Standard: Version 2.0. In: Proceedings of the 8th International Workshop on Satisfiability Modulo Theories (2010)

[10] Beckert, B., Nestler, B., Kiefer, M., Selzer, M., Ulbrich, M.: Experience Report: Formal Methods in Material Science. CoRR **abs/1802.02374** (2018)

[11] Benz, F., Hildebrandt, A., Hack, S.: A Dynamic Program Analysis to Find Floating-Point Accuracy Problems. In: Programming Language Design and Implementation (PLDI) (2012)

[12] Beyer, D.: Advances in automatic software verification: Sv-comp 2020. In: Tools and Algorithms for the Construction and Analysis of Systems (TACAS) (2020)

[13] Blanchet, B., Cousot, P., Cousot, R., Feret, J., Mauborgne, L., Miné, A., Monniaux, D., Rival, X.: A Static Analyzer for Large Safety-Critical Software. In: Programming Language Design and Implementation (PLDI) (2003)

[14] Boldo, S., Clément, F., Filliâtre, J.C., Mayero, M., Melquiond, G., Weis, P.: Wave Equation Numerical Resolution: A Comprehensive Mechanized Proof of a C Program. Journal of Automated Reasoning **50**(4) (2013)

[15] Boldo, S., Filliâtre, J.C., Melquiond, G.: Combining Coq and Gappa for Certifying Floating-Point Programs. In: Intelligent Computer Mathematics (2009)

[16] Boldo, S., Melquiond, G.: Flocq: A Unified Library for Proving Floating-Point Algorithms in Coq. In: IEEE Symposium on Computer Arithmetic (ARITH) (2011)

[17] Brain, M., Tinelli, C., Rümmer, P., Wahl, T.: An Automatable Formal Semantics for IEEE-754 Floating-Point Arithmetic. In: IEEE Symposium on Computer Arithmetic (ARITH) (2015)

[18] Brain, M., Schanda, F., Sun, Y.: Building Better Bit-Blasting for Floating-Point Problems. In: Tools and Algorithms for the Construction and Analysis of Systems (TACAS) (2019)

[19] Chapman, R., Schanda, F.: Are We There Yet? 20 Years of Industrial Theorem Proving with SPARK. In: Interactive Theorem Proving (ITP) (2014)

[20] Chen, L., Miné, A., Cousot, P.: A Sound Floating-Point Polyhedra Abstract Domain. In: Asian Symposium on Programming Languages and Systems (APLAS) (2008)

[21] Chiang, W.F., Gopalakrishnan, G., Rakamaric, Z., Solovyev, A.: Efficient Search for Inputs Causing High Floating-point Errors. In: Principles and Practice of Parallel Programming (PPoPP) (2014)

[22] Cimatti, A., Griggio, A., Schaafsma, B., Sebastiani, R.: The MathSAT5 SMT Solver. In: Proceedings of Tools and Algorithms for the Construction and Analysis of Systems (TACAS) (2013)

[23] Cok, D.R.: OpenJML: JML for Java 7 by extending OpenJDK. In: NASA Formal Methods (2011)

[24] Cordeiro, L.C., Kesseli, P., Kroening, D., Schrammel, P., Trtík, M.: JBMC: A Bounded Model Checking Tool for Verifying Java Bytecode. In: Computer Aided Verification (CAV) (2018)

[25] Cuoq, P., Kirchner, F., Kosmatov, N., Prevosto, V., Signoles, J., Yakobowski, B.: Frama-C. In: Software Engineering and Formal Methods (SEFM) (2012)

[26] Darulova, E., Izycheva, A., Nasir, F., Ritter, F., Becker, H., Bastian, R.: Daisy - Framework for Analysis and Optimization of Numerical Programs. In: Tools and Algorithms for the Construction and Analysis of Systems (TACAS) (2018)

[27] Darulova, E., Kuncak, V.: Towards a Compiler for Reals. TOPLAS **39**(2) (2017)

[28] De Moura, L., Bjørner, N.: Z3: An Efficient SMT Solver. In: Tools and Algorithms for the Construction and Analysis of Systems (TACAS) (2008)

[29] Eilers, M., Müller, P.: Nagini: A Static Verifier for Python. In: Computer Aided Verification (CAV) (2018)

[30] Filliâtre, J.C., Paskevich, A.: Why3 — Where Programs Meet Provers. In: European Symposium on Programming (ESOP) (2013)

[31] Fox, A., Harrison, J., Akbarpour, B.: A Formal Model of IEEE Floating Point Arithmetic. HOL4 Theorem Prover Library (2017), https://github.com/HOL-Theorem-Prover/HOL/tree/master/src/floating-point

[32] Fumex, C., Marché, C., Moy, Y.: Automating the Verification of Floating-Point Programs. In: Verified Software: Theories, Tools, and Experiments (VSTTE) (2017)

[33] Gao, S., Kong, S., Clarke, E.M.: dReal: An SMT Solver for Nonlinear Theories over the Reals. In: Automated Deduction – CADE-24 (2013)

[34] Ge, Y., de Moura, L.: Complete Instantiation for Quantified Formulas in Satisfiabiliby Modulo Theories. In: Computer Aided Verification (CAV) (2009)

[35] Goubault, E., Putot, S.: Static Analysis of Finite Precision Computations. In: Verification, Model Checking, and Abstract Interpretation (VMCAI) (2011)

[36] Goubault, E., Putot, S.: Robustness Analysis of Finite Precision Implementations. In: Asian Symposium on Programming Languages and Systems (APLAS) (2013)

[37] Harel, D., Kozen, D., Tiuryn, J.: Dynamic Logic. In: Handbook of Philosophical Logic, pp. 99–217. Springer (2001)

[38] Harrison, J.: Floating Point Verification in HOL Light: The Exponential Function. Formal Methods in System Design **16**(3) (2000)

[39] IEEE, C.S.: IEEE Standard for Floating-Point Arithmetic. IEEE Std 754-2008 (2008)

[40] Izycheva, A., Darulova, E., Seidl, H.: Counterexample and Simulation-Guided Floating-Point Loop Invariant Synthesis. In: Static Analysis Symposium (SAS) (2020)

[41] Jacobs, B., Smans, J., Philippaerts, P., Vogels, F., Penninckx, W., Piessens, F.: VeriFast: A Powerful, Sound, Predictable, Fast Verifier for C and Java. In: NASA Formal Methods (NFM) (2011)

[42] Jacobsen, C., Solovyev, A., Gopalakrishnan, G.: A Parameterized Floating-Point Formalizaton in HOL Light. Electronic Notes in Theoretical Computer Science **317** (2015)

[43] Jeannet, B., Miné, A.: Apron: A Library of Numerical Abstract Domains for Static Analysis. In: Computer Aided Verification (CAV) (2009)

[44] Lam, M.O., Hollingsworth, J.K., Stewart, G.W.: Dynamic Floating-point Cancellation Detection. Parallel Comput. **39**(3) (2013)

[45] Leavens, G.T., Baker, A.L., Ruby, C.: Preliminary design of JML: A behavioral interface specification language for Java. ACM SIGSOFT Software Engineering Notes **31**(3) (2006)

[46] Leavens, G.T., Cheon, Y.: Design by Contract with JML (2006), http://www.jmlspecs.org/jmldbc.pdf

[47] Leino, K.R.M.: This is Boogie 2 (June 2008), https://www.microsoft.com/en-us/research/publication/this-is-boogie-2-2/

[48] Magron, V., Constantinides, G., Donaldson, A.: Certified Roundoff Error Bounds Using Semidefinite Programming. ACM Trans. Math. Softw. **43**(4) (2017)

[49] Marché, C., Paulin-Mohring, C., Urbain, X.: The KRAKATOA tool for certification of Java/JavaCard programs annotated in JML. The Journal of Logic and Algebraic Programming **58**(1) (2004)

[50] McCormick, J.W., Chapin, P.C.: Building High Integrity Applications with SPARK. Cambridge University Press (2015)

[51] Meyer, B.: Applying “Design by Contract”. Computer **25**(10) (1992)

[52] Moscato, M., Titolo, L., Dutle, A., Muñoz, C.: Automatic Estimation of Verified Floating-Point Round-Off Errors via Static Analysis. In: SAFECOMP (2017)

[53] Muller, J., Brisebarre, N., de Dinechin, F., Jeannerod, C., Lefèvre, V., Melquiond, G., Revol, N., Stehlé, D., Torres, S.: Handbook of Floating-Point Arithmetic. Birkhäuser (2010)

[54] Müller, P., Schwerhoff, M., Summers, A.J.: Viper: A Verification Infrastructure for Permission-Based Reasoning. In: Verification, Model Checking, and Abstract Interpretation (VMCAI) (2016)

[55] Pasareanu, C.S., Mehlitz, P.C., Bushnell, D.H., Gundy-Burlet, K., Lowry, M.R., Person, S., Pape, M.: Combining unit-level symbolic execution and system-level concrete execution for testing NASA software. In: International Symposium on Software Testing and Analysis (ISSTA) (2008)

[56] Siegel, S.F., Mironova, A., Avrunin, G.S., Clarke, L.A.: Using Model Checking with Symbolic Execution to Verify Parallel Numerical Programs. In: International Symposium on Software Testing and Analysis (ISSTA) (2006)

[57] Solovyev, A., Jacobsen, C., Rakamaric, Z., Gopalakrishnan, G.: Rigorous Estimation of Floating-Point Round-off Errors with Symbolic Taylor Expansions. In: Formal Methods (FM) (2015)

